# AGuIX^®^ from bench to bedside—Transfer of an ultrasmall theranostic gadolinium-based nanoparticle to clinical medicine

**DOI:** 10.1259/bjr.20180365

**Published:** 2018-08-29

**Authors:** François Lux, Vu Long Tran, Eloïse Thomas, Sandrine Dufort, Fabien Rossetti, Matteo Martini, Charles Truillet, Tristan Doussineau, Guillaume Bort, Franck Denat, Frédéric Boschetti, Goran Angelovski, Alexandre Detappe, Yannick Crémillieux, Nathalie Mignet, Bich-Thuy Doan, Benoit Larrat, Sébastien Meriaux, Emmanuel Barbier, Stéphane Roux, Peter Fries, Andreas Müller, Marie-Caline Abadjian, Carolyn Anderson, Emmanuelle Canet-Soulas, Penelope Bouziotis, Muriel Barberi-Heyob, Céline Frochot, Camille Verry, Jacques Balosso, Michael Evans, Jacqueline Sidi-Boumedine, Marc Janier, Karl Butterworth, Stephen McMahon, Kevin Prise, Marie-Thérèse Aloy, Dominique Ardail, Claire Rodriguez-Lafrasse, Erika Porcel, Sandrine Lacombe, Ross Berbeco, Awatef Allouch, Jean-Luc Perfettini, Cyrus Chargari, Eric Deutsch, Géraldine Le Duc, Olivier Tillement

**Affiliations:** 1 NH TherAguix SAS, Villeurbanne, France; 2 Univ Lyon Université Claude Bernard Lyon 1, CNRS, Institut Lumière Matière, LYON, France; 3 Nano-H SAS, Saint-Quentin-Fallavier, France; 4 Imagerie Moléculaire In Vivo, Inserm, CEA, CNRS, Univ Paris Sud, Université Paris Saclay - Service Hospitalier Frédéric Joliot, Orsay, France; 5 Institut de Chimie Moléculaire, Université de Bourgogne, Dijon, France; 6 CheMatech, Faculté des Sciences Mirande, Dijon, France; 7 MR Neuroimaging Agents, Max Planck Institute for Biological Cybernetics, Tuebingen, Germany; 8 Dana-Farber Cancer Institute, Harvard Medical School, Boston, Massachusetts, MA, USA; 9 Centre de Résonance Magnétique des Systèmes Biologiques, CNRS UMR, Université Bordeaux, Bordeaux, France; 10 Chimie ParisTech, PSL Research University, Unité de Technologies Chimiques et Biologiques pour la Santé (UTCBS), Paris, France; 11 CNRS, UTCBS UMR , Paris, France; 12 Université Paris Descartes Sorbonne-Paris-Cité, Paris, France; 13 INSERM, UTCBS U 1022, Paris, France; 14 NeuroSpin, CEA Saclay, Gif-sur-Yvette, France; 15 Université Paris-Saclay, Orsay, France; 16 INSERM, Univ. Grenoble Alpes, Grenoble Institut des Neurosciences , Grenoble, France; 17 Institut UTINAM, UMR CNRS 6213-Université de Bourgogne Franche-Comté, Besançon, France; 18 Clinic of Diagnostic and Interventional Radiology, Saarland University Medical Center, Homburg/Saar, Germany; 19 Department of Medicine, University of Pittsburgh, Pittsburgh, Pennsylvania, USA; 20 Department of Radiology, University of Pittsburgh, Pittsburgh, Pennsylvania, USA; 21 Univ Lyon, CarMeN Laboratory Institut National de la Santé et de la Recherche Médicale U1060,INRA U1397, Université Lyon 1, INSA Lyon, Oullins, France; 22 Institute of Nuclear & Radiological Sciences & Technology, Energy & Safety, National Center forScientific Research "Demokritos", Aghia Paraskevi, Athens, Greece; 23 Université de Lorraine, CNRS, CRAN, Nancy, France; 24 Laboratoire Réactions et Génie des Procédés, UMR, Université de Lorraine-CNRS, Nancy, France; 25 Radiotherapy department, CHU de Grenoble, Grenoble cedex 9, France; 26 Department of Radiology and Biomedical Imaging, University of California, San Francisco, San Francisco, USA; 27 UNIV Lyon - Université Claude Bernard Lyon 1, Villeurbanne, France; 28 Hospices Civils de Lyon, plateforme Imthernat, Hôpital Edouard Herriot, Lyon, France; 29 Centre for Cancer Research and Cell Biology Queen's University Belfast,, Belfast BT9 7AE, UK; 30 IPNL, PRISME, Laboratoire de Radiobiologie Cellulaire et Moléculaire, Faculté de Médecine Lyon-Sud, Université Lyon 1; Hospices Civils de Lyon, Centre Hospitalier Lyon-Sud, Pierre-Bénite, France; 31 ISMO UMR, Université Paris Saclay, Université Paris Sud, CNRS, Orsay cedex, France; 32 Cell death and Aging team, Gustave Roussy, rue Edouard Vaillant, Villejuif, France; 33 Laboratory of Molecular Radiotherapy INSERM, Gustave Roussy, rue Edouard Vaillant, Villejuif, France; 34 Gustave Roussy, rue Edouard Vaillant, Villejuif, France; 35 Université Paris Sud - Paris , rue Edouard Vaillant, Villejuif, France; 36 French Military Health Academy, Ecole du Val-de-Grâce, Paris, France; 37 Institut de Recherche Biomédicale des Armées, Bretigny-sur-Orge, France; 38 Radiotherapy Department, Gustave Roussy, Villejuif, France; 39 Brachytherapy Unit, Gustave Roussy Cancer Campus, Villejuif, France

## Abstract

AGuIX^®^ are sub-5 nm nanoparticles made of a polysiloxane matrix and gadolinium chelates. This nanoparticle has been recently accepted in clinical trials in association with radiotherapy. This review will summarize the principal preclinical results that have led to first in man administration. No evidence of toxicity has been observed during regulatory toxicity tests on two animal species (rodents and monkeys). Biodistributions on different animal models have shown passive uptake in tumours due to enhanced permeability and retention effect combined with renal elimination of the nanoparticles after intravenous administration. High radiosensitizing effect has been observed with different types of irradiations *in vitro* and *in vivo* on a large number of cancer types (brain, lung, melanoma, head and neck…). The review concludes with the second generation of AGuIX nanoparticles and the first preliminary results on human.

## Introduction

Radiosensitization by nanomaterials has attracted significant interests in the last decade. In this context, our team has developed a novel radiosensitizing nanoparticle (NP) named AGuIX^®^ that underwent extensive pre-clinical evaluation and was recently translated to Phase I evaluation in the clinic for the treatment of brain metastases and advanced cervical cancer.^1^ In this paper, we summarize the preclinical evidence that has supported the transfer of AGuIX to first in man clinical evaluation.

Radiotherapy (RT) is one of the main treatment options for patients suffering from cancer. Indeed, more than 50% of all cancer patients receive RT during their treatment. In some types of cancer such as breast or central nervous system (CNS) tumours, the utilization of RT can reach 80–90% of patients.^2^ Advances in RT have focussed on improving the positioning and precision of radiation fields to the tumour target and reducing the consequential toxicities caused by irradiation of surrounding organs at risk.^3^ Contemporary RT techniques, such as intensity-modulated radiation therapy, volumetric-modulated arc therapy and image-guided radiation therapy (IGRT) have significantly improved tumour targeting,^4^ while another potential approach to improve therapeutic index is the use of radiosensitizers. This approach has been extensively explored over the past 40 years^5^ but more recently high atomic number (Z) NPs such as gadolinium (Z = 64),^6^ hafnium (Z = 72),^7^ platinum (Z = 78),^8^ gold (Z = 79)^9^ or bismuth (Z = 82) have been investigated.^10^ These elements, which are high electron emitters,^11^ have the capacity to act as radiosensitizers and amplify the effects of radiation when activated by photons of keV to MeV energies, electrons, neutrons or fast ions (>50 MeV amu^–1^).^12, 13^ Effective radiosensitization by elements such as gold has been observed at concentrations as low as 10 µg gold/g body weight in mice,^11^ although these effects cannot be explained by macroscopic dose enhancement and are not fully understood yet.^14^ It has been proposed that nanoscale dose deposition leads to important formation of radical species in the vicinity of the NPs.^15^ Despite a significant number of pre-clinical studies reports demonstrating the potential efficacy, only two NPs have been translated to clinical radiosensitizers, namely NBTXR3^7^ developed by Nanobiotix company (Paris, France), an hafnium-based intratumorally administered NP, and AGuIX developed by NH TherAguix company (Lyon, France), a gadolinium-based intravenously administered NP.^16^

Nanomedicine research is a relatively new field of research and innovation that has attracted much attention.^17^ However, despite significant research efforts, less than 100 nanomedicine based drugs have progressed to the market since the acceptance of liposomal doxorubicin in 1995.^17^ Challenges in translation to the clinic are mainly due to (i) difficulties in industrialization of synthesis and cGMP processes, (ii) non-suitable pharmacokinetics with long retention in the body and low penetration in the tumours for largest nanomedicines, (iii) eventual toxicity. Most nanomedicines are soft (organic) NPs (liposomes, polymeric NPs…) designed for improvement of drug delivery especially in the field of oncology.^18^ In contrast, inorganic NPs display advantageous properties that widen their utilization as for instance magnetic and optical hyperthermia,^19^ medical imaging such as MRI for superparamagnetic compounds or CT for metallic core^20^ and radiosensitization.^21^ Currently, only iron oxide NPs have been used as contrast agents for MRI in the clinic (Endorem^®^, GastroMARK™, Resovist^®^), but most of them have been withdrawn from the market.^22^ Another superparamagnetic iron oxide agent (Feraheme^®^) is on the market but it is dedicated to the treatment of iron deficiency anaemia.^23^

The translation of inorganic NPs to the clinic is relatively new and complicated. These NPs can be administered locally in the case of (i) NBTXR3 (hafnium-based NPs) for prostate cancer, head and neck cancer, liver cancer, soft tissue sarcoma or rectum cancer^7^ or (ii) Nanotherm^®^ (iron oxide NPs) developed by MagForce for the treatment of glioblastoma by magnetic hyperthermia^24^ or intravenously such as (i) AuroShell (large core (silica) shell (gold) NPs of 155 nm) developed by NanospectraBioscience for photothermal ablation of tumours,^25^ (ii) CYT-6091 (Pegylated gold NPs) developed by CytImmune designed for drug delivery,^26^ (iii) NU-0129 (gold NPs) developed by the Northwestern University and designed for nucleic acids delivery,^27^ (iv) Cornell dots (fluorescent polysiloxane nanoparticles) developed by Wiesner et al as an optical-PET imaging probe for melanoma^28^ and (v) AGuIX NPs (nanoparticles made of polysiloxane and gadolinium chelates) for improving MRI diagnosis of solid tumours together with their treatment by radiation therapies (RT and particle therapy).^29^

Two of these inorganic nanoparticles (AGuIX and Cornell Dots), are ultrasmall nanostructures (hydrodynamic diameter <10 nm) that allow rapid renal elimination to avoid long retention in the body and eventual toxicity after intravenous (i.v.) administration.^30^ Larger systems such as AuroShell^®^ have demonstrated a long residence time in organs including the liver and spleen after i.v. administration (>1 year), and even in the absence of toxicity in animal models, this could be a challenge for further development.^25^ Moreover, studies have emphasized the higher efficacy of ultrasmall nanoparticles to penetrate tumours, thanks to accumulation by the enhanced permeability and retention effect [enhanced permeability and retention (EPR) effect].^31^ However, these insights have been shown on animal models and have to be confirmed on humans where the EPR effect is known to be heterogeneous.^32^

**Figure 1.**
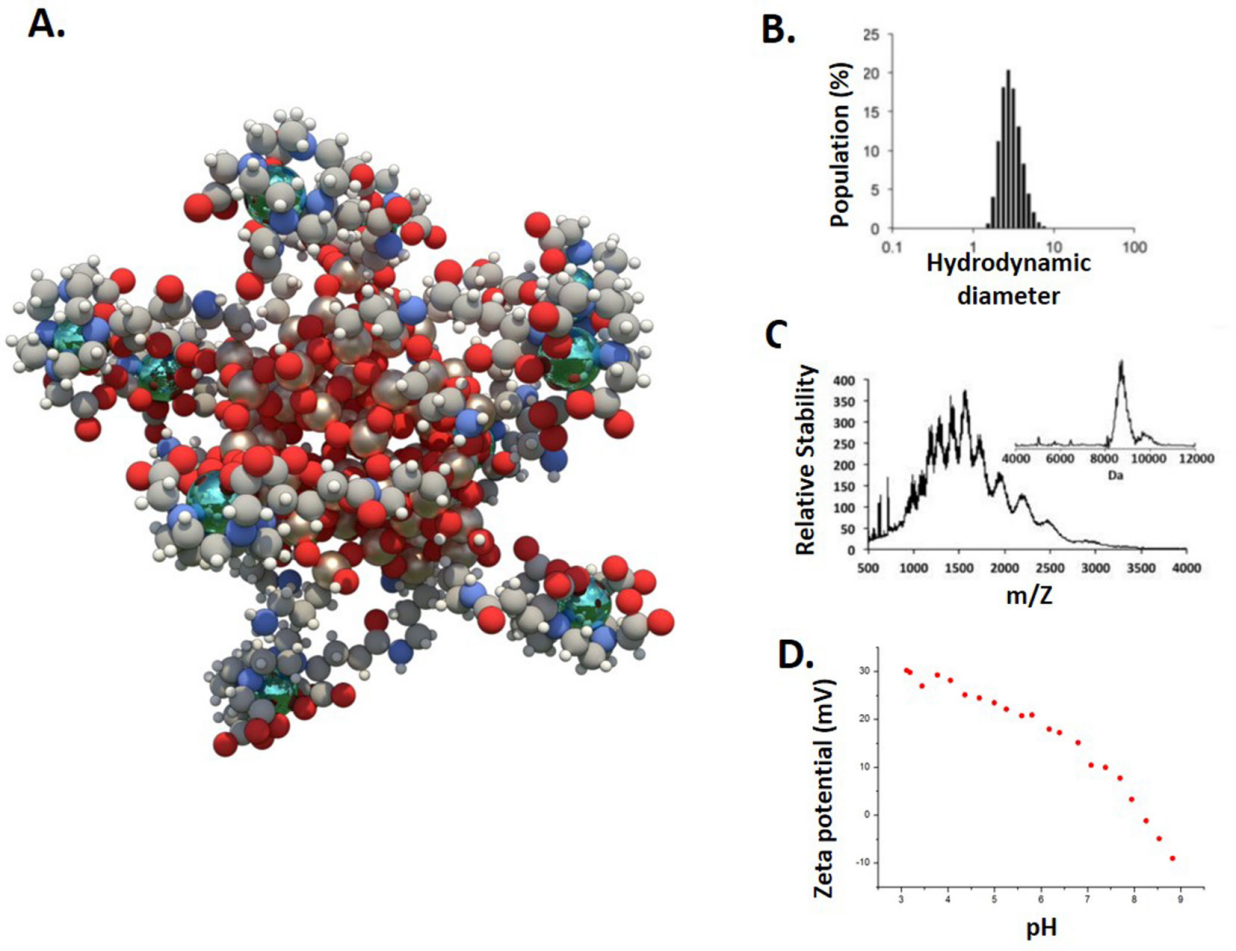
(A) Schematic representation of AGuIX^®^ NPs (gadolinium atoms in green are chelated in DOTAGA ligands grafted to polysiloxane matrix). (B) Hydrodynamic diameter (~3 nm) distribution of AGuIX NPs as obtained by dynamic light scattering. (C) ESI-MS measurements on AGuIX nanoparticles. A mass around 10 kDa is obtained for the particle. Inset is obtained after using deconvolution with a multiplicative correlation algorithm. (D) Zeta potential *vs* pH for AGuIX NPs. Adapted from.^30 ^NPs, nanoparticles.

AGuIX are sub-5 nm nanoparticles that were firstly described in 2011.^33^ They are composed of a polysiloxane matrix with gadolinium cyclic chelates covalently grafted on the inorganic matrix (Figure 1).^33, 34^ AGuIX NPs have demonstrated very high radiosensitizing properties^1^ together with excellent MRI positive contrast properties thanks to the paramagnetic properties of gadolinium.^35^ A review on the preclinical results obtained with AGuIX technology was published in 2014 highlighting the most relevant preclinical evidence at that time.^1^Since then, the synthesis process has been improved, regulatory toxicity tests have been performed and two clinical trials have been accepted by the French regulatory office ANSM (Agence Nationale de Sécurité du Médicament et des produits de santé):^36^

- NanoRAD (NCT02820454) for the treatment of brain metastases by whole brain radiation therapy in association with AGuIX NPs at Grenoble Alpes University Hospital.
- NanoCOL (NCT03308604) for the treatment of locally advanced cervical cancer by chemoradiation and brachytherapy in association with AGuIX NPs at Institut Gustave Roussy, Villejuif.

The object of the present review is to focus on the new developments of the AGuIX nanoplatform and its translation to the clinic.

## Biodistribution and pharmacokinetic

### Biodistribution and toxicity studies in healthy animals

AGuIX NPs display sub-5 nm hydrodynamic diameters, which enables renal elimination.^37^ Several biodistribution studies have been performed on healthy animals of different species (rodents and monkeys) under different conditions including: different administration methods, different administered concentrations and times of animals sacrifice after administration to better understand the behaviour of AGuIX NPs in the body.

**Figure 2.**
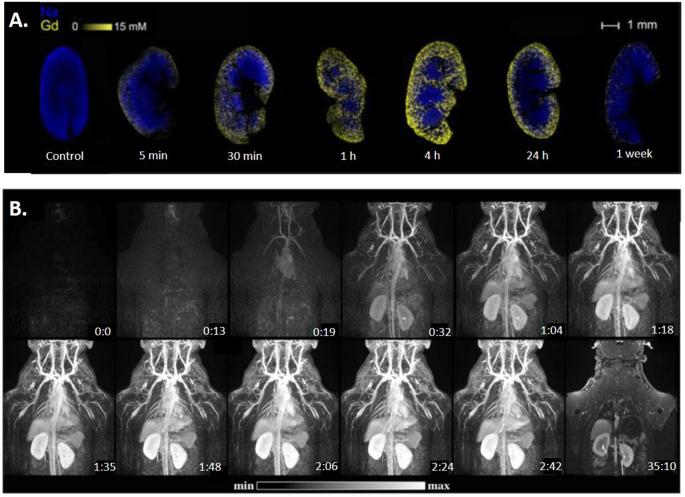
(A) Laser-induced breakdown spectroscopy analysis (Gd and Na) after i.v. administration (8 µmol) in mice. (B) MRI first pass kinetics of AGuIX NPs in a male monkey after iv injection of AGuIX NPs. Adapted from.^30^^,^^38^ NPs, nanoparticles.

To verify that AGuIX NPs are effectively eliminated by the kidneys, AGuIX NPs have been labelled by ^111^In and administered intravenously in mice and animals were sacrificed 3 and 24 h after administration. In both cases, less than 0.15% of the injected dose is observed in organs other than kidneys and bladder.^33^ A more specific study on rodents using complementary techniques was performed to study the route of elimination.^30^ AGuIX NPs (8 µmol in gadolinium/animal) were administered intravenously to mice, which were then sacrificed at different time points and kidneys collected, epoxy-embedded and sliced for imaging by laser-induced breakdown spectroscopy (Figure 2A). This experiment allows mapping and quantification of inorganic elements in biological samples.^39^ NPs were observed in the kidneys 5 min after administration and localised close to the cortex region. The maximum Gd signal was observed 4 h after administration while almost no signal could be obtained 1 week later. In other experiments, AGuIX NPs were labelled with rhodamine and injected intravenously. The observation of the kidneys by intravital two-photon microscopy analysis, performed 5 min after administration, indicated that the NPs were observed in the renal blood vessels. Tubules were reached 1 h after administration and the signal remained detectable after 1 and 48 h. Only a weak signal was observed in some of the tubules 1 week after administration and was no longer visible 2 weeks later. Renal function was monitored by measuring the serum creatinine levels. A transient increase of the serum creatinine level was observed 30 min after administration most likely due to bolus injection.

In parallel to this study, AGuIX NPs were administered intravenously in cynomolgus monkeys and pharmacokinetics was monitored by MRI (Figure 2B).^38^ The same pattern of biodistribution was observed for rodents. The vascular network and main organs (*i.e.* kidneys, heart and liver) were clearly identified. Haemodynamic, cardiac and ventilation parameters were monitored and no influence of bolus injection was detected. A large fraction of the nanoparticles was eliminated from the blood during the first 30 min and could be detected in the ureter as early as 150 s after administration. At the administered dose of 200 mg kg^−1^ (1 mg of AGuIX NPs corresponds approximately to 1 µmol of gadolinium), MRI images indicated a blood half-life of around 2 h. These two studies confirmed that renal elimination is the mechanism of AGuIX NPs clearance and that there is almost no uptake in other organs.

Regulatory toxicity and pharmacokinetics studies were performed in compliance with good laboratory practice on two animal species: rats (Wil Research company) and Cynomolgus monkeys (Wil Research company). In a dose response study, rats (16 animals/group) received two repeated i.v. injections (once a week during 2 weeks) at 250, 500 and 750 mg kg^−1^. For this evaluation, nanoparticles were formulated at pH 7.2 and dispersed in water 1 h prior to injection. No treatment-related clinical signs were observed during the follow-up period (14 days) and no hypersensitivity reactions were reported, even after multiple injections at the highest doses. Dose escalation studies have also been performed in Cynomolgus monkeys, 3 doses were tested (150, 300 and 450 mg kg^−1^; 6 animals/group). A protocol consisting of twice repeated i.v. injection protocol was used (*i.e.* once a week during 2 weeks).^38^ No adverse clinical signs were observed during the treatment period. AGuIX NPs were well-tolerated and did neither affect neurological function nor cardiac and respiratory rates. Again, no hypersensitivity reaction was reported (absence of any cutaneous reaction, even at sites of injection). Only transient vacuolations of the renal tubules have been reported only in rodents, similarly to gadolinium chelates. The study with rodents indicated a no observable adverse effect level corresponding to an human equivalent dose of 120 mg kg^−1^ approximately. In Cynomolgus monkeys, the 450 mg kg^−1^ dose corresponds to the no observable effect level. These studies have been used to determine the dose of AGuIX NPs administered during phase Ib clinical trials NanoRAD and NanoCOL that will determine AGuIX NPs tolerance in humans in association with RT and chemotherapy (NanoCOL for cisplatin).

### Biodistribution and toxicity studies after i.v. administration in tumour-bearing animals

**Figure 3.**
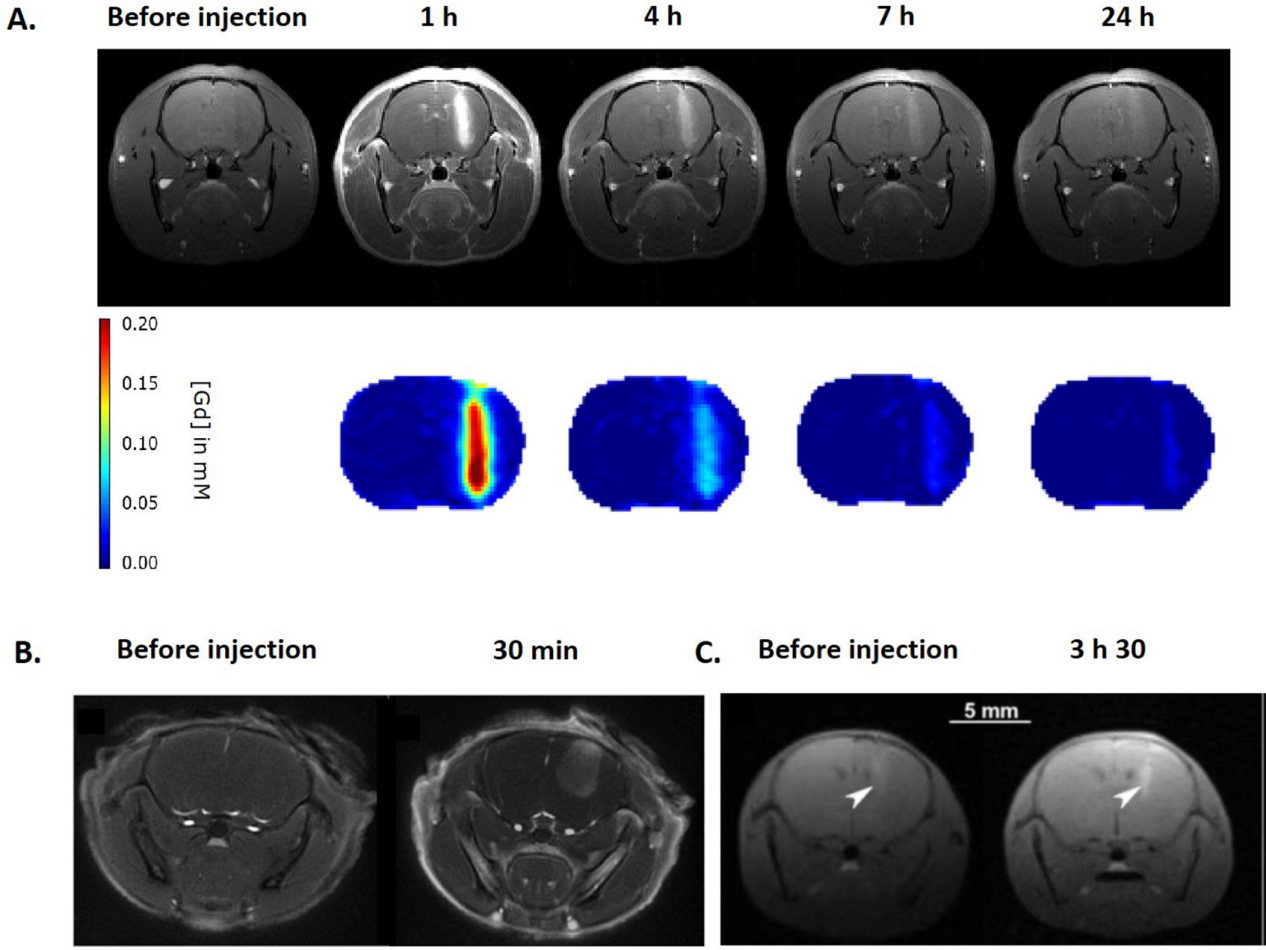
Accumulation of AGuIX NPs in tumours of the CNS. (A) MRI and gadolinium quantification at different time points after administration of AGuIX NPs in 9L-gliosarcoma-bearing rats. (B) MR axial image of the brain before and after intravenous administration of AGuIX NPs in U87MG tumour bearing mice. (C) T_1_ weighted image of the brain of a B16F10 tumour-bearing mouse after administration of AGuIX NPs. Adapted from,^16, 29^and.^41^ CNS, central nervous system; NPs, nanoparticles.

Due to the presence of gadolinium, AGuIX NPs provide MRI positive contrast agent properties. The observed longitudinal relaxivities rates are two to three times higher than clinically approved Gd-based contrast agents. MRI properties give insights into the pharmacokinetics and uptake in tumours.^34^ AGuIX NPs have been tested on different orthotopic tumour models of the CNS: (i) 9L gliosarcoma bearing rats,^29, 40^ (ii) U87MG glioblastoma bearing mice^41^ and (iii) B16F10 brain melanoma metastases bearing mice (Figure 3).^16^ In each case, tumour uptake was observed. It is due to the EPR effect. Interestingly, a retention time in the tumour significantly longer than the time of the molecular agents like DOTAREM^®^ was observed.^42^ Indeed, particles were still detected by MRI 24 h after their i.v. administration. This pharmacokinetic is fully adapted to clinical transfer since RT protocols include fractionation of radiation treatments over several days such as those used in NanoRAD trial (10 sessions of 3 Gy each for a 2 weeks duration). No evidences of AGuIX NPs extravasation in healthy brain tissue was observed. NPs were rapidly eliminated from the circulation, avoiding potential damages to healthy tissue, especially in the case of whole brain treatment where both tumour and normal tissue were irradiated.

To obtain more quantitative biodistribution data of AGuIX NPs, PET/MRI experiments were performed on U87MG heterotopic tumour bearing mice. AGuIX NPs were labelled with two different isotopes: a short-lived (^68^Ga, half-life 68 min)^43^ and a long-lived (^89^Zr, half-life 78 h)^44^ to study differential pharmacokinetics. Before radiolabelling, AGuIX NPs were functionalized by ligands known to have high affinity for the two isotopes (NODAGA for ^68^Ga and DFO for ^89^Zr). Radiolabelled AGuIX NPs displayed the same pattern of biodistribution. A rapid renal elimination resulting in extremely low background activity in all the tissues other than kidneys was observed. Further study with ^68^Ga showed an important increase of tumour-to-muscle ratio from 2.06 to 4.40 and 4.71 at 30, 60 and 120 min post-injection (p.i.) respectively, indicating retention of labelled NPs in the tumour but elimination from the circulation.^43^ AGuIX NPs are biodegradable in diluted medium.^45^ A metabolites study performed by high-performance liquid chromatography showed some degradation fragments only in urine, in contrast to blood and tumours where only entire nanoparticles were detected.^43^

After radiolabelling with ^89^Zr, tumour-associated radioactivity was evaluated after 24 and 72 h,^44^ and compared with molecular ^89^Zr-DFO. At 24 hours p.i., the molecular agent displayed a signal in the tumour four times weaker than labelled AGuIX NPs (0.5 and 2.0% ID/g respectively). Moreover, the AGuIX@^89^Zr tumour to muscle ratio increased from 4 (24 h p.i.) to values higher than 10 after 72 h, thus validating the long-term retention of the NPs in the tumours.

The residence time of the NPs in brain tumours of 9LGS bearing rats was quantified by performing X-ray fluorescence of the tissues 1 and 24 h after i.v. administration.^40^ The presence of gadolinium was assessed and quantified by L-α emission line at 6.0 keV and the quantity of AGuIX NPs in tumours found was close to 5.5 and 0.15 ppm at 1 and 24 h after i.v. administration respectively.

**Figure 4.**
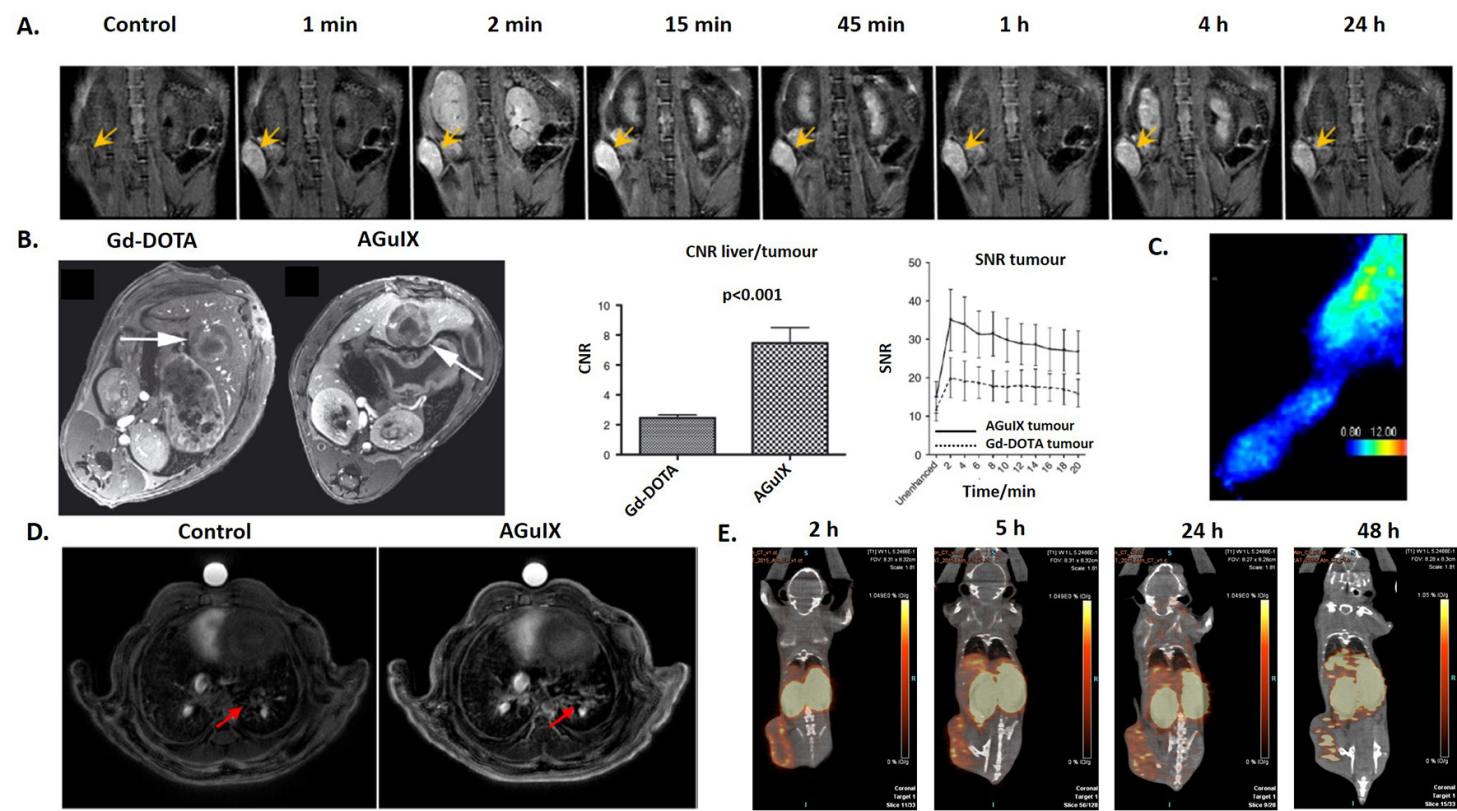
(A) *T*_1_ weighted MRI of a pancreatic tumour bearing mouse after i.v. administration of AGuIX NPs. Yellow arrows indicate tumour localization. (B) *T*_1_ weighted image of hepatic colorectal cancer metastasis after i.v. administration of molecular agent (Gd-DOTA) and AGuIX NPs. Comparison of contrast-to-noise ratio and signal-to-noise ratio for Gd-DOTA and AGuIX NPs in tumour. (C) *In vivo* SPECT imaging in the paw of a swarm rat chondrosarcoma orthotopic model after i.v. administration of radiolabelled ^111^In AGuIX NPs. (D) Ultrashort echo-time MRI axial slices of a lung tumour bearing mouse before and after i.v. administration of AGuIX NPs. (E) PET/CT imaging of a 4T1 tumour bearing mice after i.v. administration of radiolabelled ^64^Cu AGuIX NPs. Adapted from,^35,46–48^. i.v., intravenous; NPs, nanoparticles; SPECT, single photon emission computed tomography.

In addition to animals bearing CNS tumours, biodistribution imaging experiments have been performed on animals with pancreatic (capan-1),^46^ colorectal (CC531),^47^ chondrosarcoma (swarm rat chondrosarcoma),^48^ lung (Luciferase modified non-small cell lung cancer H358)^35^ and breast (4T1) tumours after i.v. administration of AGuIX NPs (Figure 4). As previously shown for tumours of the CNS, rapid tumour uptake was observed with a long retention time. Indeed, the NPs were still detected 24 h after i.v. administration in pancreatic,^46^ lung^49^ and breast cancer (Figure 4).

For example, a study was performed on mice bearing breast 4T1 tumours using AGuIX NPs functionalized with NODAGA and ^64^Cu (life time of 12.7 h). The signal in the tumour was relatively stable from 5 to 48 h after injection (at around 2% ID g^–1^) confirming the long retention. As mentioned in the case of CNS tumours, long term retention is an important factor during fractionated RT protocols used in clinic.

### Alternative administration routes of AGuIX^®^ NPs

**Figure 5.**
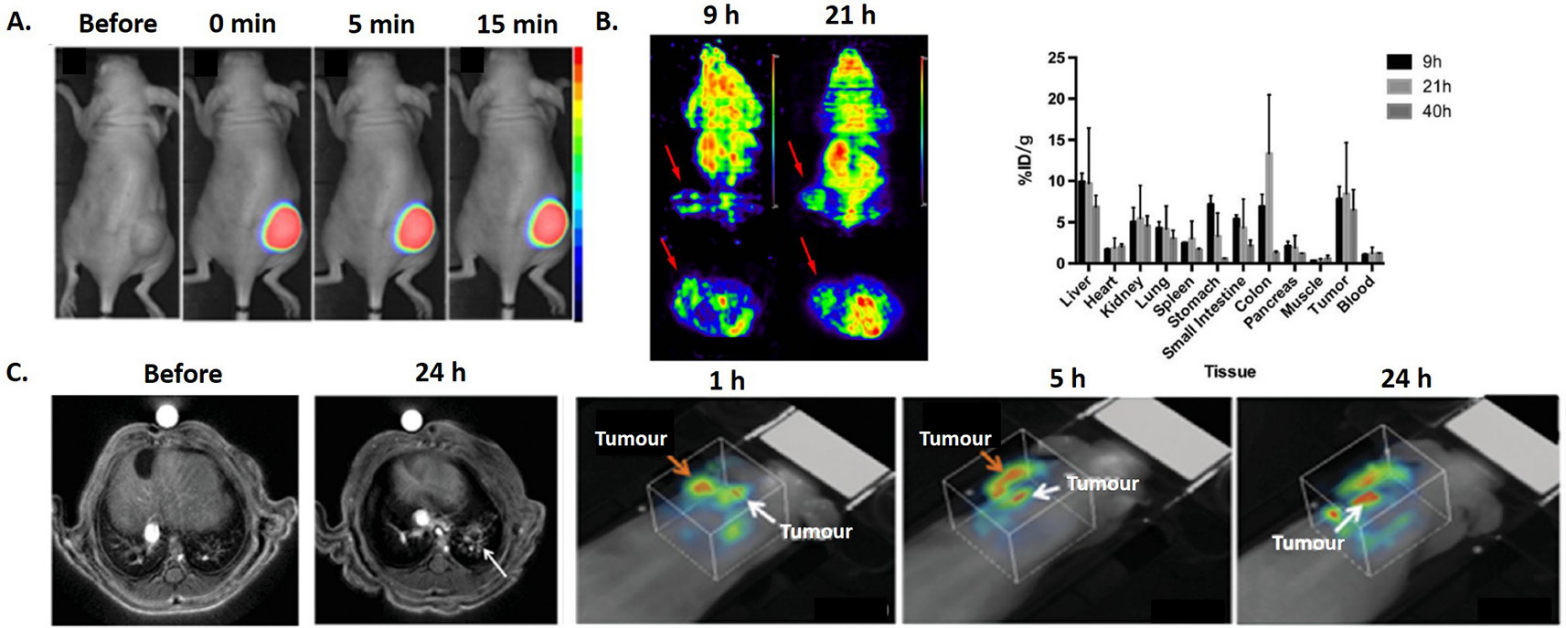
(A) Optical images of a head and neck SQ20B tumour bearing mouse after intratumoral administration of Cy 5.5 labelled AGuIX NPs. (B) Micro-PET images and biodistribution of HepG2 tumour bearing mice after intraperitoneal administration of radiolabelled ^64^Cu AGuIX NPs. Animals were sacrificed 9 h, 21 h and 40 h after intraperitoneal administration of the NPs and radioactivity was quantified. (C) MRI and 3D optical images at different time points after administration via the airways of AGuIX NPs labelled by Cy 5.5 in orthotopic NSCLC tumour bearing mice. Adapted from.^49–51^ ^50 ^3D, three-dimensional; PET, positron emission tomography.

Although i.v. administration is the preferred route of administration for translation to the clinic, other routes have been tested with AGuIX NPs (Figure 5). Like for other inorganic nanomedicines (NBTXR3 and Nanotherm^®^), intratumoral (i.t.) administration of AGuIX NPs has also been explored.^50^ This route of administration can be used for highly accessible tumours and has the advantage to deliver the exact quantity of NPs to the tumour volume, which requires smaller quantities of material. AGuIX NPs have the advantage of being dispersible at very high concentration (500 g l^−1^), which allows delivery of large quantities in very small volumes. For example, i.t. administration of 1 µmol of AGuIX NPs (in gadolinium) labelled by Cy 5.5 has been performed in nude mice bearing SQ20B tumours (Figure 5A). The whole tumour is fluorescent directly after administration and no change of the signal is observed for the first 15 min after administration. However, for translation to the clinic, additional studies are needed to demonstrate that this type of administration leads to a sufficiently homogeneous distribution of the NPs in the tumour and leakage of AGuIX NPs from the tumour to blood is likely to happen.

Another potentially interesting local administration is intraperitoneal (i.p.) delivery as recently shown by an independent study of Hu et al in a hepatocellular carcinoma model^51^ (Figure 5B). The biodistribution of the AGuIX NPs has been assessed after radiolabelling by ^64^Cu and animals sacrificing 9, 21 and 40 h after i.p. administration. Accumulation with high retention in the tumour was observed, with uptakes of 7.82 ± 1.50, 8.43 ± 6.23 and 6.84 ± 1.40% ID g^–1^ at 9, 21 and 40 h respectively and with no significant accumulation in other normal tissues (except kidneys and bladder).

Our team has also studied administration *via* the airways in mice where we observed rapid passage from the lung tissue into blood circulation after intratracheal administration of NPs which was followed by renal elimination.^52^

An MRI pharmacokinetic study showed that the elimination half-time from the lungs is of the order of 130 ± 20 min independently of the administered NPs concentration (10, 25 and 50 mM in [Gd^3+^]).^53^ This value is significantly higher than the one obtained for DOTAREM (22 ± 5 min; 250 mM in [Gd^3+^]) and give access to a larger imaging window. The relatively rapid elimination observed from the lungs for AGuIX NPs (hydrodynamic diameter <5 nm) is in good agreement with the previous study of Choi et al with quantum dots.^54^ Moreover, a short term toxicological study was performed to evaluate adverse effects through this route of administration. In this preliminary study, no significant change in the number of inflammatory cells or in pathological changes in the alveolocapillary barrier were observed. These results further support translation to the clinic of AGuIX NPs.

When administered to orthotopic lung tumour bearing mice (Figure 5C),^34^ retention in the tumour was observed allowing non-invasive detection of millimetre-size tumours with excellent correlation between MRI signal, bioluminescence and histology. Using four times less gadolinium than i.v. administration (200 µl, [Gd^3+^] = 50 mmol l^−1^), administration *via* the airways (50 µl, [Gd^3+^] = 50 mmol l^−1^) resulted in two times higher values of signal enhancement and an increase in contrast-to-noise ratio. Higher signal in the tumour for the airways delivery can be explained by rapid passage of the NPs from the healthy tissues of the lung to the blood circulation and specific retention in the tumour tissue. Besides, after passage into the bloodstream, the NPs can be taken up by EPR effect. In the case of lung tumours, administration by airways may be more interesting since lower amounts of NPs are needed to reach therapeutic concentrations in the tumour. As illustrated in the orthotopic U87 glioblastoma study, this mode of administration may also be used to improve tumour diagnosis and treatment of other organs since NPs go to the circulation and concentrate in tumours via EPR effect.^40^

This novel delivery method can also be applied to image pathologies other than cancer such as idiopathic pulmonary fibrosis, a chronic, progressive and ultimately lethal disease.^55^ In this study, idiopathic pulmonary fibrosis has been induced by intratracheal administration of bleomycin that results in excessive fibroblast activation and extracellular matrix proliferation. Without contrast agent, MRI fails to delineate clearly the induced lesions but after intratracheal administration of AGuIX NPs, all lesions are clearly detected and delineated in animals. The elimination times of the NPs from the lungs were determined by MRI. As observed in two different models (C57BL/6 and BALB/c), longer elimination times were measured for injured animals (250.6 ± 22 and 249.7 ± 26.1 min) in comparison with healthy animals (166.3 ± 6.3 and 159.6 ± 13.6 min). These different elimination rates allow injured lung to be distinguished from healthy tissue. More generally, studying the pharmacokinetics and elimination of AGuIX NPs in the lungs may contribute to the detection of lung injuries.

**Figure 6.**
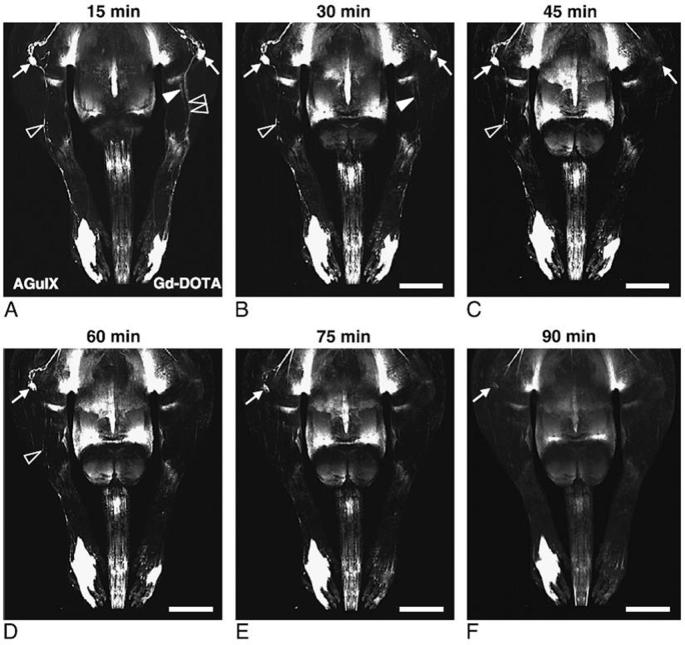
Comparison of lymphadenography with AGuIX NPs and Gd-DOTA. Scale bars: 9.5 mm. Copyright from.^56 ^NPs,

AGuIX NPs have also been recently tested for magnetic resonance lymphography (MRL) after intradermal injection on a chronic lymphedema model of the rat hindlimb.^56^ In addition to imaging of the lymphatic vessels, MRL can give access to lymph node characterization. Contrary to most of the other compounds tested for MRL (dendrimers, dextran, hepatobiliary contrast agents…), AGuIX NPs do not exhibit delayed renal elimination and uptake into the mononuclear phagocyte system. In this study, AGuIX NPs were injected intradermally in the left hindlimb and Gd-DOTA (DOTA: 1,4,7,10-Tetraazacyclododecane-1,4,7,10-tetraacetic acid) as a control (Figure 6). Both contrast agents highlighted the popliteal lymph node with enhancing signal as early as 15 min after administration. AGuIX NPs administration was associated with an about two times higher signal-to-noise ratio in comparison with molecular Gd-DOTA. In contrast to Gd-DOTA, signal enhancement did not drop to baseline but persisted up to 90 min after administration. Moreover, due to a higher hydrodynamic diameter (around 3 nm), AGuIX NPs were mostly drained from the injection site into the lymphatic vessels. Contrary to AGuIX NPs behaviour, Gd-DOTA was drained both by the short saphenous vein and lymphatic vasculature resulting in images being more difficult to analyse by the overlaying venous enhancement. This study emphasizes the potential interest of AGuIX NPs for MRL after reconstructive lymphedema surgery or for lymph node detection.

## Radiosensitization

**Figure 7.**
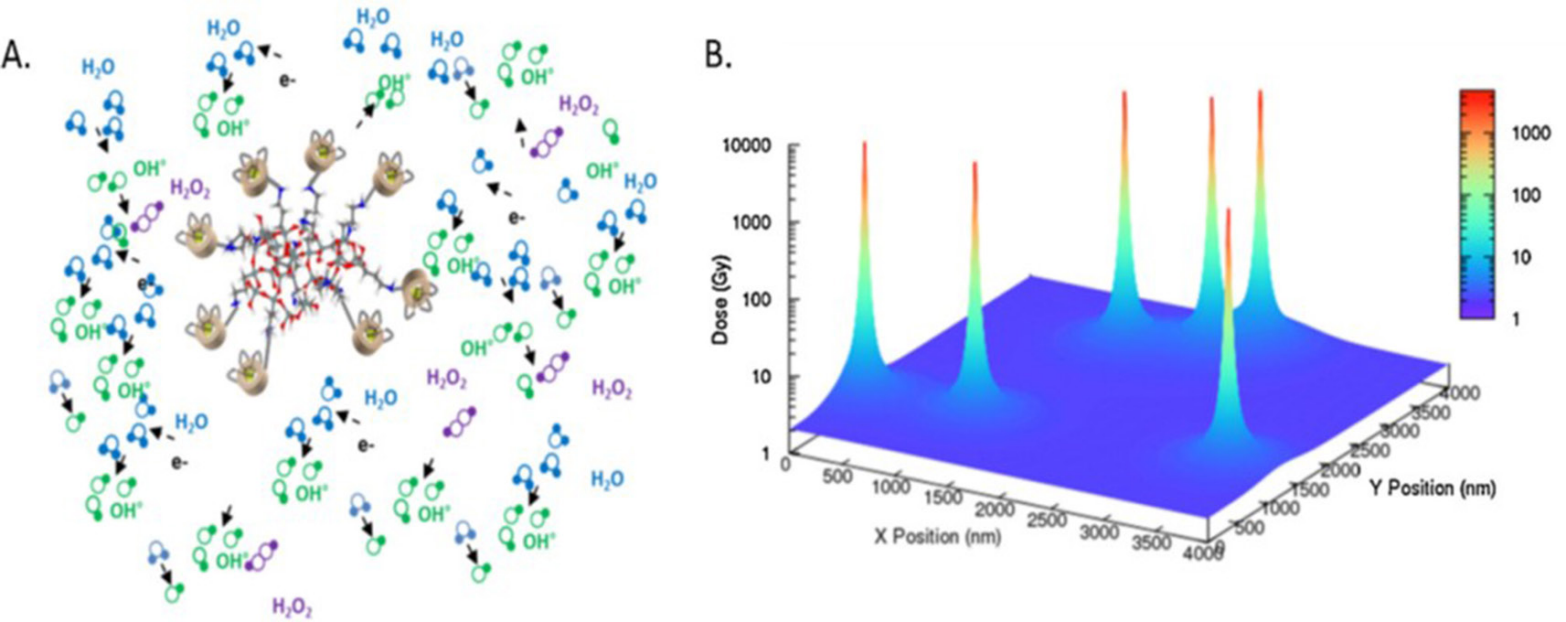
(A) Schematic formation of electron showers and reactive oxygen species obtained after irradiation. (B) Simulation of the deposited dose after irradiation at 2 Gy. Adapted from.^57^

Although AGuIX NPs provide excellent imaging, they were initially developed as radiosensitizers in the context of image-guided radiotherapy. As previously reviewed,^1^ AGuIX NPs have shown radiosensitizing efficacy *in vitro* on a various number of cell lines with typical sensitizer enhancement ratios varying from 1.1 to 2.5 for photon irradiation at different energies ranging from keV to MeV. Sensitizer enhancement ratios are defined as the survival fraction ratios for the control cells (irradiation alone) to those of the treated cells (irradiation combined with nanoparticles). This radiosensitizing effect cannot be explained by macroscopic dose enhancement alone. In addition, modelling of the radiosensitization can be explained by nanoscale dose deposition in the vicinity of the nanoparticles. Such modelling has been performed after irradiation at 80 keV (Figure 7).^57^ The clustering of a few gadolinium atoms leads to the formation of an Auger shower inducing a strong increase of the deposited dose close to the gadolinium cluster. However, more theoretical studies are needed to more clearly determine the exact mechanism of action of AGuIX NPs in association with RT. Correlating MRI enhancement and local clinic response will be of particular importance to answer this point during clinical studies.

### In vitro

**Figure 8.**
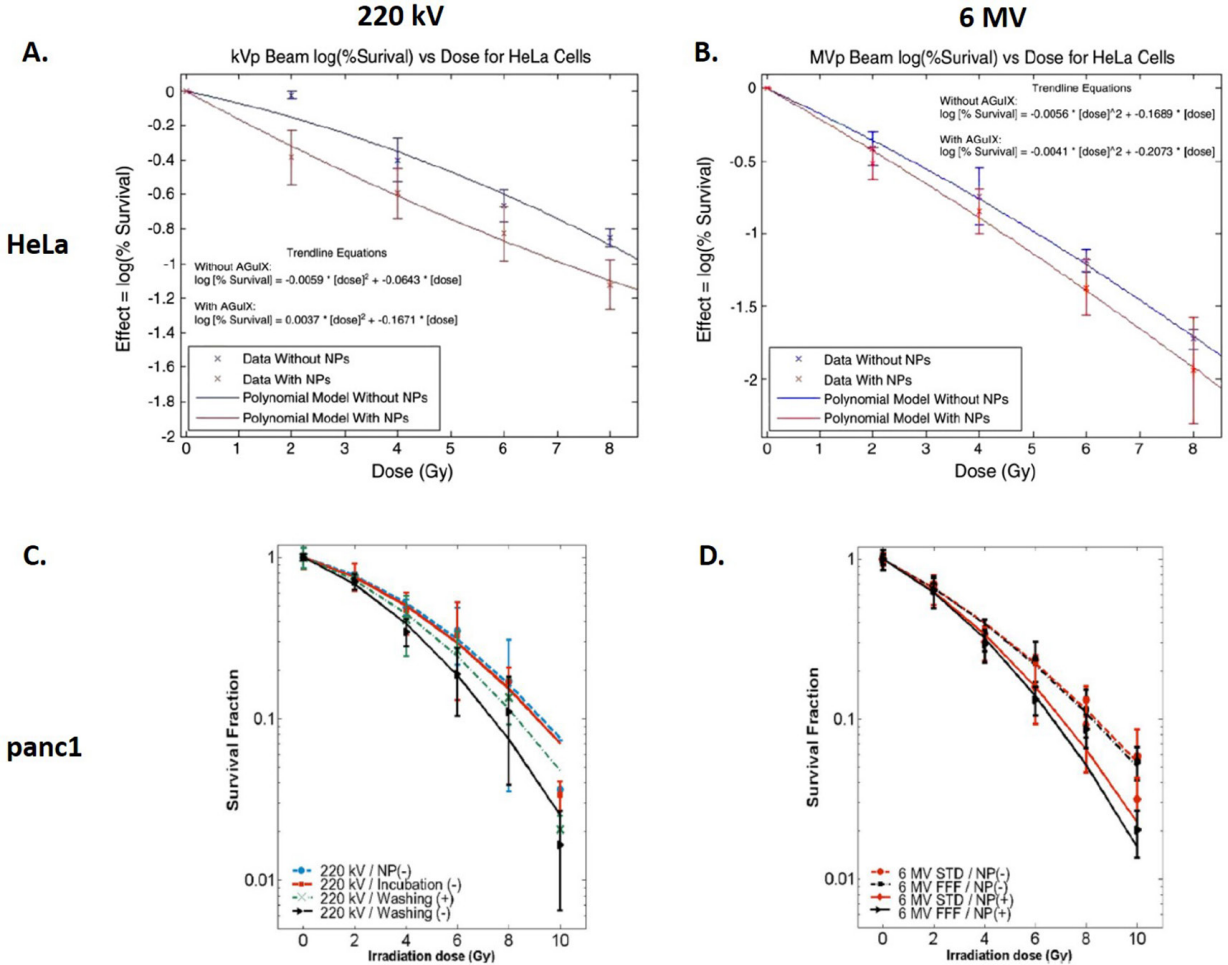
Survival of HeLa cells (A, B) or panc1 cells (C, D) after irradiation with 220 kV (A, C) or 6 MV (B, D) with or without AGuIX NPs at different doses. Adapted from^59^ and.^60^

In particular, the radiosensitizing effects of AGuIX NPs on clinical irradiators at 6 MV have been demonstrated in a number of cell lines including cervical carcinoma – HeLa (incubation at [Gd^3+^] = 0.5 mM) (SER_4Gy_ = 1.3; Dose Enhancement Factor (DEF) = 1.2),^57^ glioblastoma–U87MG (SER from 1.10 to 1.50 for concentrations ranging from 0.1 to 0.5 mM)^58^ and pancreatic adenocarcinoma–panc1 (DEF = 1.30) (incubation at [Gd^3+^] = 0.5 mM) (Figure 8).^46, 59^ The DEF is the ratio of the area between the survival curves with and without NPs.

### New mechanisms for radiotherapy in association with AGuIX NPs

Investigations have been performed to better understand cell death mechanism of RT in combination with AGuIX NPs.

A recent study in radioresistant head and neck squamous cell carcinoma (HNSCC) SQ20B cells aimed to understand the radiosensitizing mechanisms in presence of AGuIX NPs.^61^ Using 250 kV X-Rays, a surviving fraction at 4 Gy of 1.382 was obtained with an enhanced biological factor of 1.3 (incubation of cells at 0.8 mM in gadolinium). According to a previous study on U87MG glioblastoma cells,^58^ AGuIX NPs are shown to principally localize in lysosomes with no particles detected in the nucleus. To demonstrate the importance of ROS production in the mechanism of AGuIX NPs radiosensitization, depletion of glutathione (GSH), an endogenous ROS scavenging system was tested on SQ20B cells. After treatment, a net increase in ROS production was observed when irradiation was combined with AGuIX NPs (incubation of cells at 0.8 mM in gadolinium) and resulted in an increase of enhanced biological factor from 1.3 to 1.6. Interestingly, autophagy and/or autophagic cell death significantly increased after irradiation in combination with AGuIX NPs.

### Radiosensitization with protons and other ions

An alternative to photon irradiation is the use of fast ion beams (70–400 MeV amu^–1^) to treat solid tumours (proton therapy and hadrontherapy when protons and carbon ions are used respectively). These irradiations are generally proposed for the treatment of tumours located in highly sensitive tissues such as eyes and brain, and also for paediatric cancers or highly radioresistant tumours.^62^ Ion irradiation offers better tumour targeting in comparison with conventional X-ray radiation due to specific high energy delivery at the end of the track (Bragg peak). This prevents damages to the healthy tissues located behind the tumour volume. The induction of low but significant damages in the healthy tissues located in front of the tumour remains a major limitation. The fast development of particle beam facilities all around the world has increased the interest in developing agents capable of improving the performances of this treatment modality. In particular, it has been demonstrated that the addition of high-Z nanoagents increases the effect of ion beam radiation.^13^ This allows to use NPs to amplify the effects in the tumour and to decrease the total dose given to the patient, so decreasing adverse effects to healthy tissues. Moreover, as for conventional RT, the use of multimodal NPs (good electron emitters also active in medical imaging modalities—MRI for instance) offers the possibility to develop “image-guided particle therapy”.

The efficiency of AGuIX NPs to amplify the effects of medical protons was demonstrated using a 150 MeV proton beam under two irradiation conditions mimicking the entrance (0.44 keV µm^–1^) and the end (3.6 keV µm^–1^) of the proton track^63^ on plasmid pBR322. In particular, it was demonstrated that the yields of complex damages (nanosize breaks lethal for the cells) is amplified for irradiation in association with AGuIX NPs. More importantly, this increase was found to be more important at the end of the proton track than at its entrance (from amplification factor_complex_ =1.19 ± 0.06 at 0.44 keV µm^–1^, to 1.32 ± 0.04 at 3.6 keV µm^–1^) (incubation of plasmids at 0.04 mM in gadolinium). When the same experiment was performed in the presence of dimethyl sulfoxide, a radical scavenger, an important decrease of the damage yield was observed, confirming the role of hydroxyl radicals in the radiosensitization associated with protons. Similar experiments were realized using He^2+^ and C^6^^+^ and led to the same conclusions as for those with photons (*i.e.* increase of lethal damages when irradiation is associated with AGuIX NPs in comparison with control groups: amplification factor_complex_ of 1.45 and 1.73 for He^2+^ and C^6^^+^ respectively). The increase is more pronounced for incident C^6+^. Like for irradiation with protons, the major role of hydroxyl radicals in the radiosensitization of particle beam treatment associated with AGuIX NPs was evidenced.

**Figure 9.**
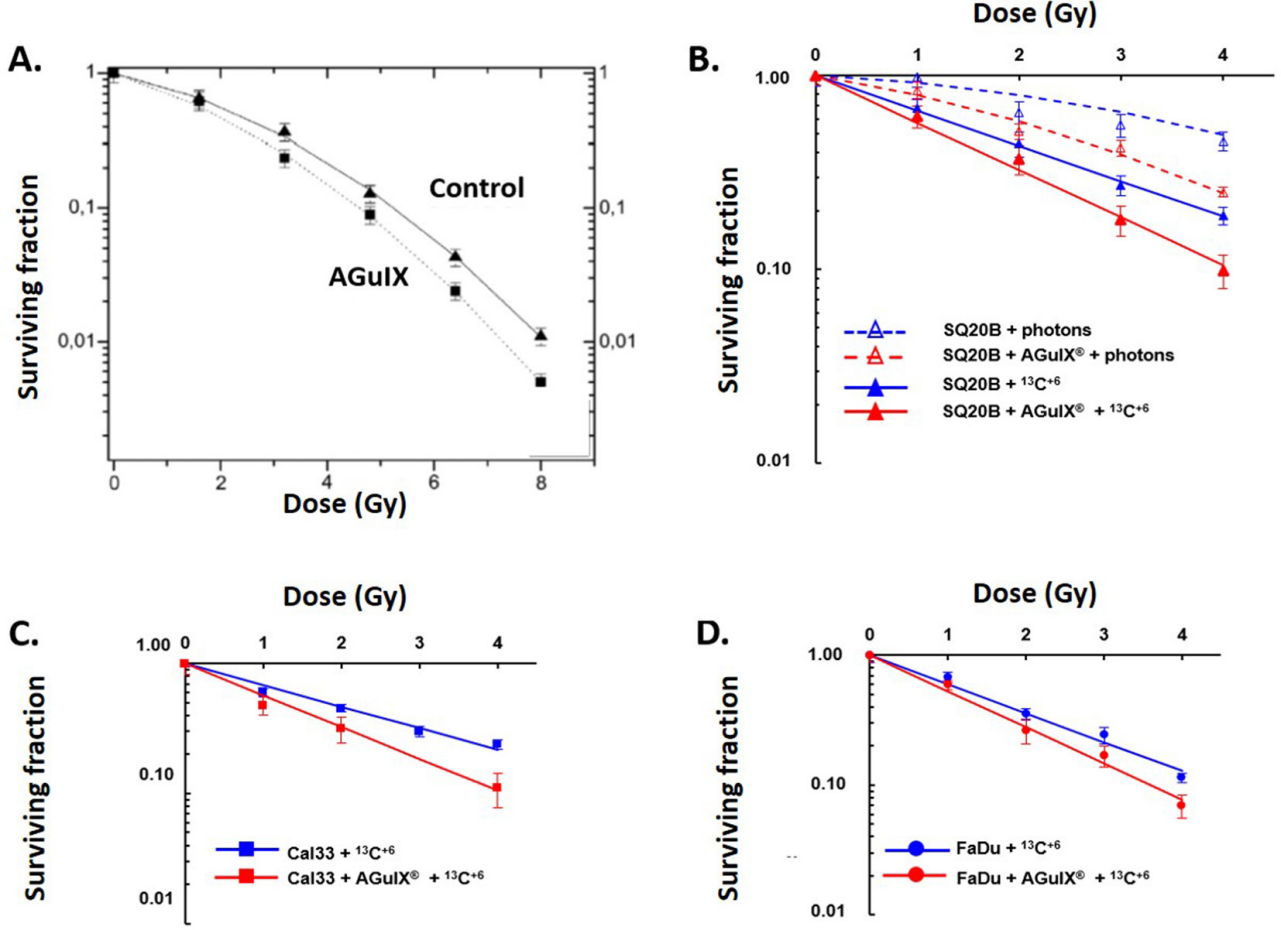
Survey of CHO (A), SQ20B (B), Cal33 (C) and FaDu (D) under irradiation by hadrons at different doses with or without AGuIX NPs. Adapted from^62^ and.^65^

In addition to nanoscale evidence of the AGuIX NPs efficiency to amplify particle beam effects, *in vitro* experiments confirmed the amplification of cell killing using various models: (i) Chinese ovary cell (CHO) line (incubation of cells at 1 mM in gadolinium)^64^ (ii) three HNSCC cell lines: SQ20B, Cal33 and FaDu (incubation of cells at 0.8 mM in gadolinium) (Figure 9).^65^ In the case of CHO cells loaded with AGuIX NPs, enhancing factors of 11.3 and 18.5% for He^2+^ and C^6^^+^ respectively were found. By fitting the survival curves by the linear quadratic law (S(D) = exp (-αD–βD^2^), it was found that AGuIX NPs did not significantly change the yield of sublethal damages (β = 0.044 with He^2+^, and 0.047 with C^6+^ respectively) but significantly increased the induction of directly lethal damages (α = 0.17 *vs* 0.22 for control and AGuIX NPs with He^2+^ respectively and α = 0.19 vs 0.27 for control and AGuIX NPs with C^6+^ respectively). Thus, the increasing of the α/β ratio demonstrates an enhancement of the radiation lethality when AGuIX NPs are associated to the treatment. These results have been confirmed by irradiation with C^6+^ of three cell lines of HNSCC known for their radioresistance. Like for CHO, the β coefficient remained nearly unchanged between the control and the cells loaded with AGuIX NPs whilst the α coefficient increased significantly (0.42* vs *0.56 for SQ20B, 0.38* vs *0.56 for Cal33 and 0.51* vs *0.64 for FaDu). Here again, an increase of the relative biological effectiveness was observed for the three cell lines when irradiation was performed in association with AGuIX NPs in comparison with irradiation alone (1.7* vs *1.27 for SQ20B, 1.7* vs *1.14 for Cal33 and 1.66* vs *1.33 for FaDu). All these data emphasize the synergistic benefits of combining particle therapies (proton therapy and hadrontherapy) with AGuIX NPs and the potential for further translation to the clinic.

### Radiosensitization in different *in vivo* models

**Figure 10.**
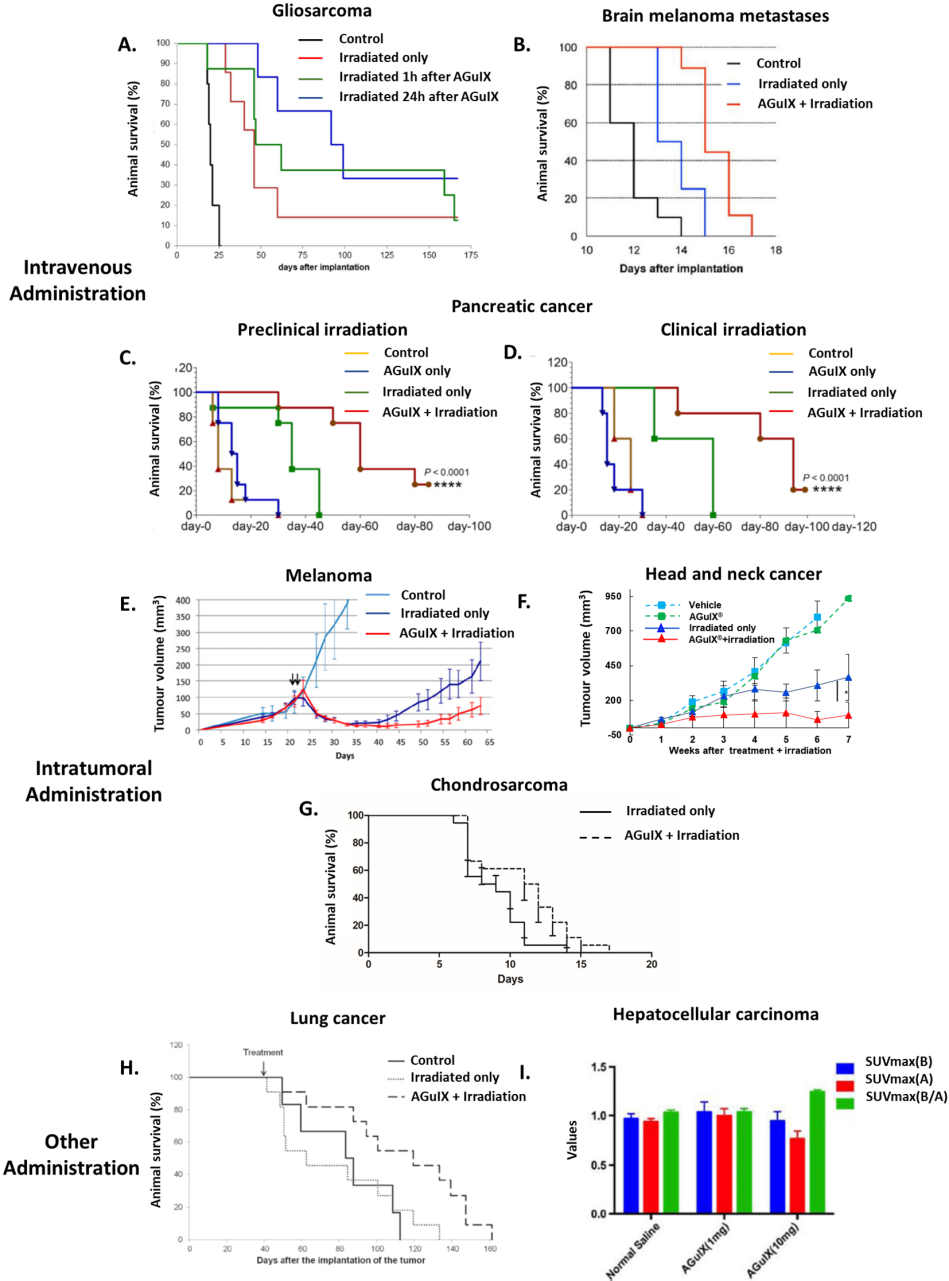
Radiosensitization on different preclinical models. (A) Survival curves obtained for 9LGS bearing rats. (B) Survival curves obtained for B16F10 brain metastases bearing mice and irradiation at 7 Gy. (C) Survival curves obtained for capan-1 tumour bearing mice under preclinical (220 kV) irradiation. (D) Survival curves obtained for capan-1 tumour bearing mice under clinical (6 MV) irradiation. (E) Relative tumour progression after intratumoral administration of AGuIX NPs in A375 tumour bearing mice. (F) Relative tumour evolution after intratumoral administration of AGuIX NPs in SQ20B tumour bearing mice. (G) Survival follow-up in a rat xenograft model of chondrosarcoma (SWARM). (H) Survival curve obtained for H358-luc tumour bearing mice after administration of AGuIX NPs by the airways. (I)^18^F-FDG PET quantitative evaluation before and after irradiation of hepatocellular carcinoma HepG2 tumour bearing mice. Adapted from,^1,16,40,46,49–51^.^18^F-18-fludeoxyglucose.

AGuIX NPs have already shown their efficiency as a radiosensitizer for different *in vivo* preclinical models of cancer (glioblastoma,^29, 33,40^ brain metastases,^16^ melanoma,^1^ pancreatic cancer,^46, 66^ liver cancer,^51^ chondrosarcoma, head and neck cancer^50^ and lung cancer^49^ ) (Figure 10). A particular attention has been paid to tumours of the CNS with studies on 9L gliosarcoma bearing rats and B16F10 melanoma brain metastases bearing mice. As previously said, after i.v. administration ([Gd^3+^] = 40 mM, 1.4 ml), a rapid uptake in the tumour was observed (as early as 5 min after administration) due to the EPR effect. Retention of the nanoparticles in the tumour for more than 24 h was assessed in brain tumour models after i.v. administration. The quantity of gadolinium in the tumour was evaluated at 5.5 and 0.15 ppm in the tumours of 9L gliosarcoma bearing rats 1 and 24 h after i.v. administration of AGuIX NPs respectively. Even if the quantity of gadolinium in the tumour was reduced at 24 h after administration, a superior mean survival time (MeST) was observed after irradiation by microbeam radiation therapy at 24 h (95.5 days) in comparison with irradiation performed 1 h after administration (62 days) or without administration of nanoparticles (46 days) (Figure 10). This corresponds to Increases in Lifespan (ILS) of 130, 210 and 377.5% for irradiation only, irradiation at 1 and 24 h after i.v. administration respectively. Interestingly, the radiosensitizing effect seemed to be more significant 24 h after administration even if the quantity of nanoparticles in the tumour is smaller. This can be explained by the fact that the nanoparticles that remained in the tumours have a particular affinity for the tumour cells and are either strongly absorbed on the cell membrane or internalized, further potentiating their radiosensitizing effect. To assess the interest of these NPs for clinical translation, an experiment was carried out using a clinical irradiator (6 MV) and the same animal model (*i.e.* 9l gliosarcoma bearing rats).^33^ In this experiment, two irradiations were performed at day 10 and day 17 after implantation of the tumours. Each of the irradiations was performed 7 h after the i.v. administration of AGuIX NPs ([Gd^3+^] = 100 mM, 1 ml). With this protocol, MeST of 26 ± 0.5 days, 39 ± 0.5 days and 72.9 ± 35.5 days were observed for the controls, the irradiation only group and irradiation in combination with AGuIX NPs.

To compare the effects of AGuIX NPs with commercial molecular gadolinium chelate (DOTAREM, Guerbet), an experiment comparing both compounds was proposed.^42^ Due to the rapid elimination of DOTAREM from the tumours, the irradiation by microbeam radiation therapy was performed 20 min after i.v. administration for each group in 9L gliosarcoma tumour bearing rats. The two groups with irradiation performed after i.v. administration of DOTAREM ([Gd^3+^] = 40 mM, 1.4 ml and [Gd^3+^] = 1 M, 56 µl) presented similar MeST (32 and 43 days) to the irradiated only group (44 days) corresponding to ILS of 68, 126 and 131%. In comparison, irradiation in association with AGuIX NPs nanoparticles ([Gd^3+^] = 40 mM, 1.4 ml) led to MeST of 102.5 days corresponding to an ILS of 439%. This result was consistent with *in vitro* experiments and simulations that emphasize the importance of the nanoparticle structure of the radiosensitizer that is responsible for not only the nanoscale dose deposition (in the very close vicinity of the NP), but also the EPR effect that is responsible for long retention of nano-objects in tumours. To assess the use of AGuIX NPs on a different model of cancer in the CNS, a study was conducted on melanoma B16F10 brain metastases tumour bearing mice.^16^ In this study, the irradiation was performed 3.5 h after i.v. administration of the nanoparticles (10 mg), when the imaging study indicated the highest quantity of gadolinium in the tumour. For this experiment, a preclinical irradiator was used (320 kV) with a single irradiation at 7 Gy. This model was very aggressive, with a MeST of 12 days for the control group, 13 days for the irradiation only group and 15 days for the irradiation in association with AGuIX NPs, corresponding to ILS of 8.3 and 25% for the irradiated group and irradiated in association with the AGuIX NPs group respectively (Figure 10). These encouraging results on two different models of brain cancer have led to the NanoRAD clinical trial (ClinicalTrials.gov Identifier: NCT02820454) that is designed to treat multiple brain metastases by whole brain radiation therapy in association with AGuIX NPs.

In parallel to these studies on tumours of the CNS, a complete study on pancreatic cancer (capan-1) was published in 2016 comparing the efficacy of AGuIX NPs in association with preclinical irradiation (220 kV) or clinical irradiation (6 MV) (Figure 10C,D).^46^ Irradiation at a dose of 10 Gy was performed 15 min after administration of AGuIX NPs (0.25 mg of AGuIX NPs g^–1^ of animal). This irradiation time was determined by MRI. This study showed the greatest signal in the tumour 15 min after i.v. administration (Figure 4). For pre-clinical irradiation, MeST of 13, 35 and 60 days were obtained for control group, irradiated only group and irradiated in association with AGuIX NPs group, corresponding to ILS of 169 and 361% for irradiated only group and combined treatment group respectively. An analysis of double strand DNA breaks after the death of the animals showed a significant increase of DNA damages for irradiation performed in combination of AGuIX NPs (~80 %) in comparison with irradiated only animals (~60 %) and control animals (<10 %). No significant DNA damages were observed for other organs like kidneys, liver, heart or lung, even if they are close to the irradiation site (liver and kidneys). Interestingly, similar efficacy was obtained for clinical irradiation with MeST of 30, 60 and 93 days for control group, irradiated only group and irradiated in association with AGuIX NPs group corresponding to ILS of 100 and 210% for irradiated only group and combined treatment group, respectively. This study was in good agreement with the study on 9L gliosarcoma bearing rats^34^ and confirmed the* in vivo* potential of AGuIX NPs with clinical irradiation.

Other studies were published using an i.t. administration of AGuIX NPs. It was tested on radiosensitive (A375 melanoma)^1^ and radioresistant (head and neck SQ20B cancer)^50^ models subcutaneously implanted on the flank of mice. For A375 melanoma tumour bearing mice, two irradiations (10 Gy each, 220 kV) were performed just after the administration of the nanoparticles (4 µmol of gadolinium) to avoid any escape of nanoparticles from the tumour. Compared with untreated mice, tumour growth delay was observed for both irradiated mice without the presence of AGuIX NPs and those irradiated in association with AGuIX NPs, with much longer delay seen for irradiation in the presence of AGuIX NPs (Figure 10E). For example, 25 days after the treatment, the tumour volume increased only by 3% when the treatment was performed after AGuIX NPs administration, but increased by 82% in absence of AGuIX NPs. For the radioresistant model (head and neck cancer), an i.t. injection (1 µmol of gadolinium) was also performed followed by an immediate irradiation (320 kV) at 10 Gy (Figure 10F). Once again, tumour growth was delayed in comparison with the irradiated only group, due to decreased cell proliferation associated with increased apoptosis. For example, at the end of Week 7, the mean tumour growth was 5 times smaller than for the irradiated only group and 11 times smaller than for the control group. Another study has been performed on radioresistant chondrosarcoma in a rat xenografted model (SWARM). 5 min after i.t. administration of 100 µl (10 mM) of AGuIX NPs, a 4 Gy irradiation was performed. A statistical significant difference (Log Rank (Mantel-Cox) test <0.02)) was observed between the two studied groups (irradiated only group and irradiated in association with AGuIX^®^ NPs group) (Figure 10G). These studies emphasize the potential of combining intratumorally administered AGuIX NPs with irradiation for the treatment of radioresistant cancers.

For some cancers, local delivery can be achieved by other types of administration. For example, delivery of nanoparticles *via* the airways demonstrated high tumour targeting capacity as shown by MRI and fluorescence imaging.^35^ To demonstrate the interest of this administration pathway for treating lung cancer, AGuIX NPs were nebulized in H358-Luc orthotopic lung tumour bearing mice ([Gd^3+^] = 40 mM, 1.4 ml). At 24 h post-administration, the animals were irradiated at 10 Gy (220 kV) (Figure 10H). No significant difference was observed between the irradiation only group and the control group, with MeST of 77 and 83 days respectively. In comparison, when the irradiation is performed in presence of AGuIX NPs, a MeST of 112 days was observed corresponding to an ILS of 35 and 45% * vs * the control group and the irradiation only group respectively. This method of administration offers the benefit of delivering a higher amount of nanoparticles in the tumour with a smaller total quantity of administered nanoparticles.

In an independent study performed by Hu et al^51^ AGuIX NPs were administered by i.p. pathway for hepatocellular carcinoma (HepG2 tumours) resulting in tumour uptake of 7.82 ± 1.50, 8.43 ± 6.23, and 6.84 ± 1.40% ID g^–1^ at 9, 21 and 40 h after administration. The animals were initially irradiated at 1 h after administration (250 kV, 6 Gy) and a second irradiation was delivered after 24 h. 1 day after this treatment, tumour metabolism was measured by 18-fludeoxyglucose positron emission tomography imaging and maximum standard uptake value were obtained in regions of interest (Figure 10I). A decrease of 18-fludeoxyglucose uptake in the tumour was observed for all the irradiated mice. Maximum standard uptake value (Before/After) of 1.03 ± 0.03, 1.04 ± 0.04 and 1.24 ± 0.02 were observed for control group and mice irradiated after administration of 1 mg and 10 mg of AGuIX NPs respectively. Thus, no significant difference was observed after administration of 1 mg of AGuIX NPs but when the administered dose is sufficient (10 mg), irradiation suppresses glucose metabolism. These two examples highlight the interest of different administration pathways as a function of the type of cancer.

To conclude on this part, AGuIX NPs have already shown their potential for combination with RT in the treatment of cancer for different animal models with a range of radiation sensitivities. Different administration pathways can be used. They include i.v. administration that only requires classical medical practice as well as local ones that permit the uptake of larger quantities of AGuIX NPs. But the latter will necessitate complementary regulatory studies for eventual clinical translation.

## New generation of aguix^®^ nanoparticles

Different modifications have been made to AGuIX NPs to develop ultrasmall polysiloxane-based NPs for improved or new applications: adding new metals on the NPs or functionalizing them with therapeutic or targeting moieties. This paper is focused on ultrasmall nanoparticles passively targeted to the tumours and therefore, studies aiming at actively targeting AGuIX NPs to tumour or other pathologies won’t be described.^67–71^ Targeted AGuIX NPs will be of great interest to increase uptake (<1% of the injected dose in animal models) and retention in the tumours.

### Addition of new metals

**Figure 11.**
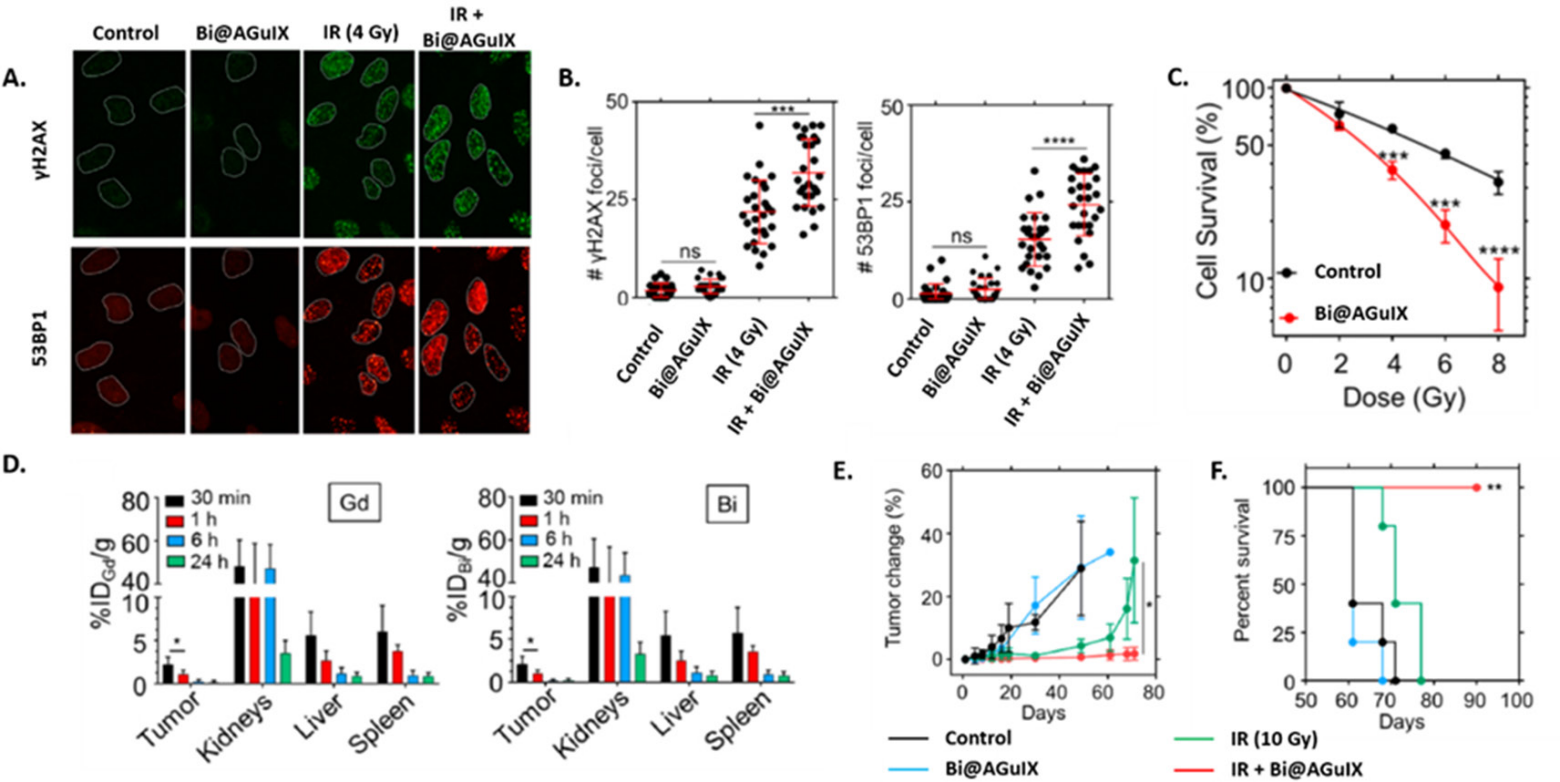
*In vitro*and *in vivo* experiments related to Bi@AGuIX NPs. (A) Qualitative visualization of γH2AX and 53BP1 foci *in vitro* irradiation of A549 adenocarcinoma cells under 6 MV irradiation. (B) Quantitative measurement of the number of γH2AX and 53BP1 foci per cell after irradiation. (C) Clonogenic assay after irradiation of A549 cells under irradiation at different doses with or without Bi@AGuIX NPs. (D) Biodistribution of Bi@AGuIX NPs after iv. administration in A549 tumour bearing mice evaluated by quantification of Gd and Bi by ICP/MS after sacrifice of the animals at different time points. (E) Evaluation of the tumour growth after treatment for A549 tumour bearing mice. (F) Survival curves obtained after the treatment A549 tumour bearing mice for the different groups. Adapted from.^10^

As shown previously for radiolabelling, ligands can be covalently grafted on AGuIX NPs to enable further chelation of metals. In the case of AGuIX NPs, gadolinium acts as a contrast agent and a radiosensitizer thanks to its high atomic number (Z = 64), but other metals having higher Z such as gold (Z = 79) and bismuth (Z = 83) present potentially a more effective interaction with irradiation.^11^ The addition of bismuth on the AGuIX NPs (Bi@AGuIX) can increase their efficacy for radiosensitization and also for CT.^10^ Bi@AGuIX NPs were obtained by grafting additional DOTA chelates on AGuIX NPs followed by the complexation of bismuth in acidic conditions. DOTA ligands were chosen due to their high affinity for Bi^3+^ (log K = 30.3).^72^ The final nanoparticles displayed a hydrodynamic diameter of 4.5 nm and around 10 DOTA chelated with Gd^3+^ and 5 DOTA chelated with Bi^3+^. Phantom measurements for MRI and CT led to a detection limit of 0.1 mg ml^−1^. At 7 T, Bi@AGuIX NPs displayed longitudinal relaxivity of 4.87 s^−1^.mM^−1^ and r_2_/r_1_ of 1.46, which is slightly higher than commercial contrast agents. For CT, a contrast of 4.26 Hounsfield unit mM^−1^ was obtained, close to those of clinically used CT contrast agents. To assess the* in vitro* radiosensitizing properties of Bi@AGuIX NPs, A549 NSCLC cells were incubated with Bi@AGuIX (incubation of cells at 0.5 mM in gadolinium) NPs 30 min before irradiation with a clinical irradiator (6 MV). When cells are incubated with Bi@AGuIX NPs, an increase in γH2AX and 53BP1 foci (Figure 11A,B) was observed associated with a significant decrease in the clonogenic survival of cells resulting in a DEF of 1.99 (Figure 11C).

After i.v. administration, the NPs were followed by CT and MRI in A549 lung adenocarcinoma tumour bearing mice. Their pharmacokinetics was assessed by sacrificing the animals 30 min, 1, 6 and 24 h after i.v. injection (Figure 11D) and quantifying Gd and Bi by ICP-MS. The results were similar for gadolinium and bismuth showing the integrity of the NPs, a rapid uptake in the tumour and an elimination via the kidneys with no significant retention in other organs. The irradiation (10 Gy, 6 MV) was performed 30 min after i.v. administration of Bi@AGuIX NPs at 420 mg kg^−^^1^ (dry weight) when the tumour uptake was maximum (3.54% ID). The two control groups showed a rapid progression of the tumour (Figure 11E) with survival more than 70 days after therapy (Figure 11F). For the irradiated group, a delay of the tumour growth was observed during the first 30 days after irradiation followed by a rapid growth of the tumour resulting in no survival 80 days after irradiation. For the group irradiated in presence of the Bi@AGuIX NPs, a clear increase in both tumour growth delay and survival was observed (Figure 11E,F). Double strand DNA breaks were evaluated 30 min after irradiation for both healthy and tumour tissues. For healthy tissues, no increase of double strand DNA breaks was observed for any groups. For the tumours, an increase of double strand DNA breaks was observed both for the irradiated only group (67.31 ± 11.36 %) and the group combining Bi@AGuIX NPs and irradiation (89.33 ± 14.3%) in comparison with both control groups (8.1 ± 4.3 and 5 ± 4.3% respectively).

As shown in this study, the addition of different metals like bismuth can provide other modalities to the AGuIX NPs without changing neither their size nor their biodistribution (*i.e.* renal elimination and tumour uptake). Other metals such as europium or terbium can also be added, thus offering fluorescent properties to the nanoparticle while radioactive isotopes can be used for PET or SPECT imaging as well as for brachytherapy.

### Functionalization by photosensitizers

The functionalization of AGuIX NPs can also provide other therapeutic modalities to the resulting nanoparticles, *e.g.* photodynamic therapy (PDT).

PDT is an alternative therapy of cancer. This technique involves the use of a photosensitizer (PS) which under illumination by light generates cytotoxic reactive oxygen species such as singlet oxygen.^73^ The first generation of PDT agents were limited by photosensitivity of the patients due to weak differentiation between healthy and diseased tissues. Improved biodistribution can be obtained by coupling the photosensitizers to nano-objects that can target the tumour by the EPR effect and/or targeting moieties and that will not extravasate into healthy tissues.^74^ In addition, to obtain an absorption of light in the near infrared region, photosensitizers are usually hydrophobic and can easily aggregate in biological media, significantly limiting the injected concentrations and modifying their photophysical properties. The use of hydrophilic nanocarriers can overcome this limitation either by protecting the photosensitizers in the core of the object or by displaying them at their surface.^75^

**Figure 12.**
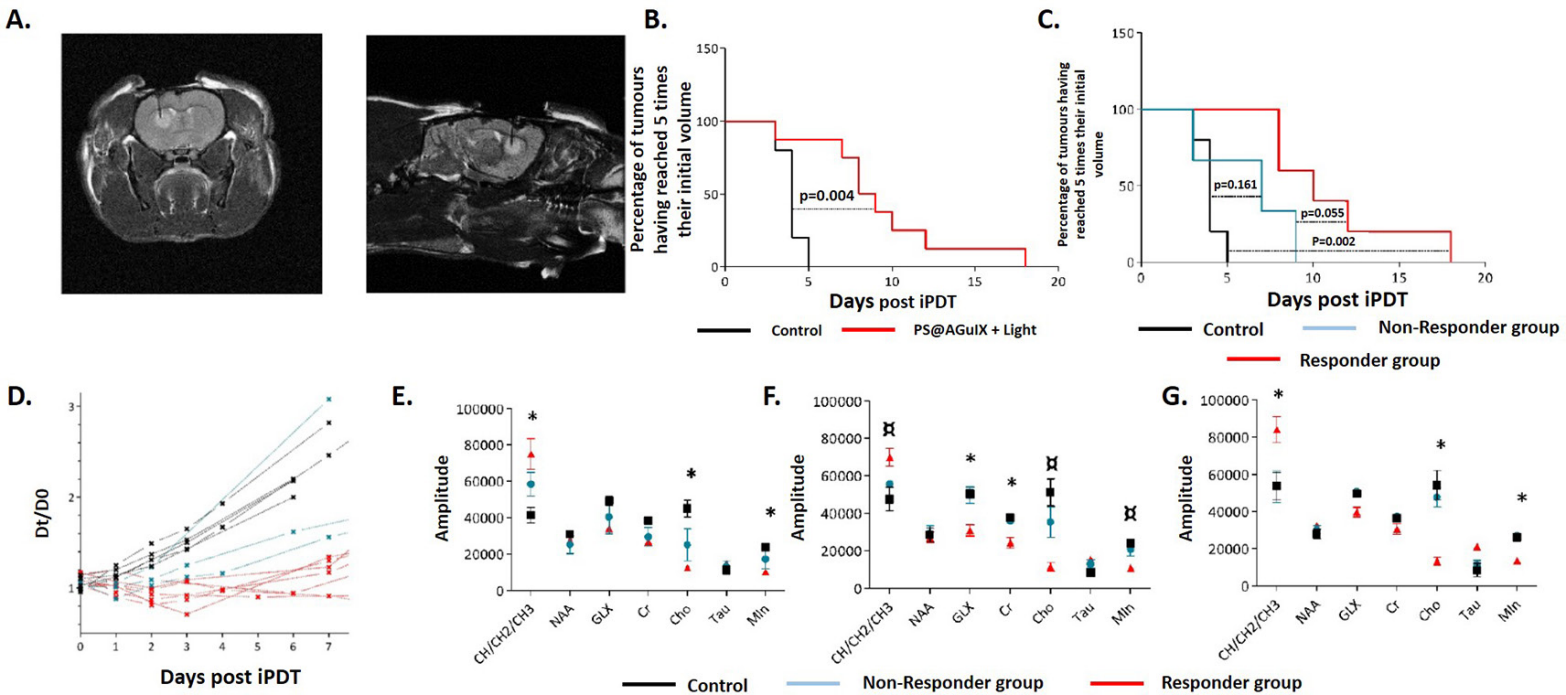
(A**) Optical fibre insertion monitored by *T*_2_ weighted MRI. (B) Survival curves obtained after illumination for control and PS@AGuIX NPs-treated groups.(C) Survival curves obtained after illumination for control, non-responder and responder groups. (D) Relative growth of the tumour diameters for the different animals and determination of non-responder and responder groups. (E) Relative levels of metabolites measured by MRS 1 day after treatment. (F) Relative levels of metabolites measured by MRS two days after treatment. (G) Relative levels of metabolites measured by MRS 3 days after treatment. Adapted from.^76 ^MRS, magnetic resonance spectroscopy; NPs, nanoparticles.

Our strategy was to graft photosensitizers on AGuIX ultrasmall NPs to obtain hydrophilic NPs displaying smaller size than most of the nanostructures developed for PDT. Moreover, the MRI contrast properties of AGuIX NPs can be useful to clearly delineate the tumour, to guide positioning of the optical fibres used in interstitial PDT (iPDT) and to optimize the drug-light interval. To demonstrate the potential of AGuIX NPs for iPDT, we have recently published a study on the treatment of glioblastoma (U87) bearing rats by iPDT in association with PS@AGuIX NPs.^76^ For this proof of concept, a well-known PS: a monocarboxylic TPP (a derivative from tetraphenylporphyrin) was grafted on the AGuIX NPs by carbodiimide chemistry. After grafting the PS, no significant changes in its photophysical properties (*i.e.* quantum yields of fluorescence and ^1^O_2_ production) were observed. After i.v. administration, tumour uptake was observed by MRI as early as 5 min after i.v. administration due to the passive targeting of the tumour by EPR effect. For light treatment, an optical fibre was placed stereotactically in the tumour of each rat and the position was confirmed by MRI (Figure 12A). The illumination of the tumour by light was performed 1 h after i.v. administration of AGuIX NPs (Figure 12B). For the treated group, inflammatory effects were observed 1 day after treatment by MRI with vasogenic and cytotoxic oedema. These inflammatory effects were not observed for the control group (*i.e.* illumination with AGuIX NPs without PS). Interestingly, for the treated group two different profiles of response were observed in the evolution of the diameter of the tumours after treatment, leading to the distinction between a responder and a non-responder group (Figure 12C,D). To explain the different tumour response between the two groups, a light dosimetry study was performed by Monte Carlo simulation based on the positioning of the optical fibre and the scattering of the photons. Based on these simulations, it was determined that only the responder group received sufficient illumination.^76^ A study of relevant intracellular metabolites was also performed by magnetic resonance spectroscopy: choline containing components (Cho), creatinine (Cr), N-acetyl aspartate, lipid such as CH/CH_2_/CH_3_ levels, myo-inositol (Min), glutamate plus glutamine, and taurine (tau). Before treatment, all groups displayed the same metabolic signature (*i.e.* comparable levels of metabolites). Just after treatment, no significant difference was observed between the three groups but interestingly differences in metabolite levels were observable as soon as one day after treatment (Figure 12E) and these differences increased during the first three days after treatment (Figure 12F,G). An increase in lipid levels was observed that is usually correlated to membrane breakdown. By contrast, a decrease of the levels of Min and Cho was observed. For Cho, the decrease of the levels was correlated with rapid cellular membrane turnover. Min is an indicator of microglial activation and proliferation while its decrease is linked to proliferation shutdown.

These data show the potential of grafting standard PS like porphyrins to ultrasmall NPs. Their pharmacokinetics after grafting to AGuIX NPs is superior to those of molecular porphyrins, with no aggregation, elimination by the kidneys and uptake in the tumours by EPR effect. Moreover, the PK can be followed by MRI enabling the detection of the tumour, the monitoring of the implantation of the optical fibre and the determination of the optimum time for illumination. However, despite these interesting results, efforts have to be made before eventual clinical translation to develop cGMP functionalization of AGuIX NPs with synthesis processes compatible with industrial specifications and limitations. Toxicity has also to be tested in GPL regulatory studies.

## First in man

**Figure 13.**
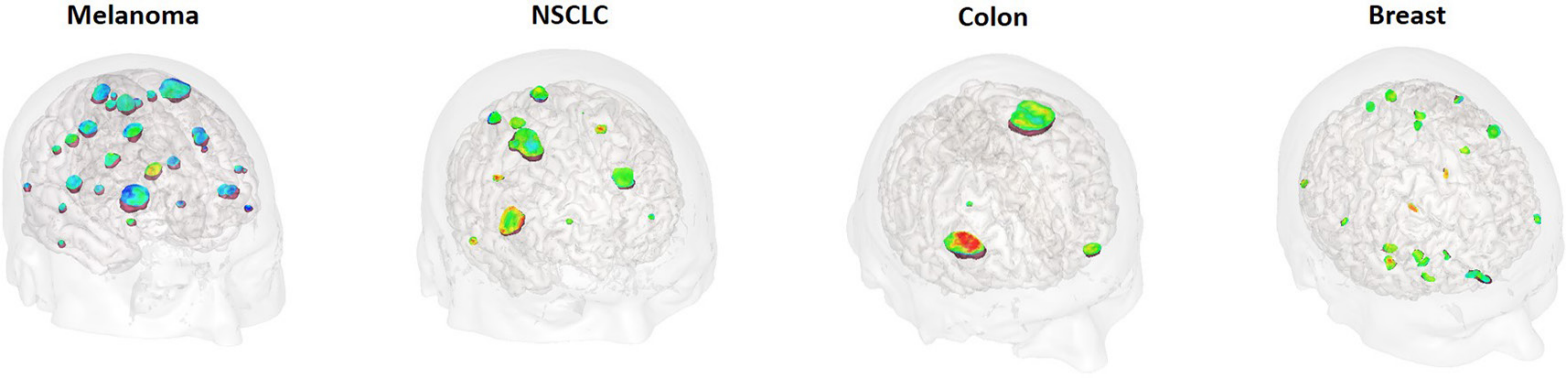
Illustration of 3D MR imaging of the NanoRAD clinical trial obtained 2 h after i.v. administration of AGuIX NPs. Brain metastases (issued from melanoma, non-small cell lung cancer NSCLC, colon cancer and breast cancer) are targeted by AGuIX NPs while no enhancement of the signal is observed in healthy tissues. NPs, nanoparticles.

The first in man administration of the AGuIX NPs was performed during the NanoRAD clinical trial (NCT02820454).^77^ NanoRAD is a phase Ib clinical trial ongoing at CHU Grenoble Alpes aimed at treating multiple brain metastases by whole brain RT (10 × 3 Gy over 3 weeks max) in combination with AGuIX Nps (one injection 4 h before the first session of RT). The NanoRAD trial was designed as a dose escalation phase Ib with five doses tested (15, 40, 50, 75 and 100 mg kg^−1^). The NPs were injected in patients with brain metastases originating from four different types of cancer: lung (NSCLC), melanoma, colon and breast. For each type of cancer, a targeting of the metastases by AGuIX NPs was observed due to the EPR effect (Figure 13). Interestingly, all the metastases already detected by conventional *T*_1_ MRI contrast agent were also detected by AGuIX NPs. To conclude, besides its strong local radioenhancing effect, one of the main properties of AGuIX nanodrug that makes it such strong candidate as a radiosensitizer is its ability to pass through the blood brain barrier selectively in brain metastases leading to the possibility of a differential response between the tumour and dose-limiting normal tissue.

**Figure 14.**
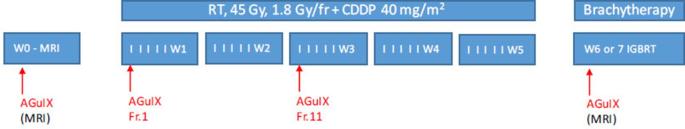
Protocol of the NanoCOL Phase Ib clinical trial.

The NanoCOL clinical trial (NCT03308604) has been accepted recently by French regulatory office. NanoCOL is a Phase Ib clinical trial ongoing in Gustave Roussy institute (Villejuif).^78^ It is aimed to treat advanced cervical cancer by RT and brachytherapy in association with AGuIX NPs and cisplatin (CDPP) (Figure 14). During the protocol, three injections of AGuIX NPs will be delivered before the first irradiation at Week 1, at the beginning of Week 3 and before brachytherapy. Conventional RT will be delivered during 5 weeks (45 Gy, 25 sessions) and concomitant weekly i.v. administration of CDPP (40 mg m^−2^) will be performed. Brachytherapy (15 Gy) will be delivered in 2 weeks. First patient of NanoCOL clinical trial has been included in May 2018.

## Conclusion

AGuIX NPs are one of the few inorganic NPs that have been translated to the clinic for theranostic applications. Besides their MRI positive contrast agent properties, they display high radiosensitizing potential at a very small concentration due to nanoscale dose deposition in the vicinity of some activated NPs. Therapeutic effect has been proven *in vivo* on eight different tumour models including radioresistant ones. One of the other key advantages of AGuIX NPs is their ultrasmall size that ensure a renal elimination avoiding uptake in the MPS after i.v. administration. For specific applications, AGuIX NPs can also be administered by more local administrations like i.t., intratracheal or i.p. administrations.

Two Phase Ib clinical trials have begun with AGuIX NPs to validate their interest for human applications, using i.v. administrations that do not disrupt the clinical workflow. One of the major question when translating an intravenously administered NP from animal models to human is their capacity to passively accumulate into tumours like observed in the models. Interestingly, MRI studies performed on the 15 patients of NanoRAD trial have shown a clear uptake in the different brain metastases of AGuIX NPs.

For the future clinical trials, other indications will be investigated. These indications will be selected depending of the possibility to increase the efficacy of the RT by increasing locally the effect of the dose without irradiating more surrounding healthy tissues.
